# DNA Fingerprint Profile of *Zizania* spp. Plant, Monitoring Its Leaves with Screening of Their Biological Activity: Antimicrobial, Antioxidant and Cytotoxicity

**DOI:** 10.3390/life15081240

**Published:** 2025-08-05

**Authors:** Latifah A. Al Shammari

**Affiliations:** Department of Pharmaceutical Chemistry, College of Pharmacy, University of Hafr Al Batin, P.O. Box 1803, Hafr Al Batin 31991, Saudi Arabia; lamalshammari@uhb.edu.sa

**Keywords:** *Zizania* spp., phytochemical profiling, antimicrobial activity, antioxidant activity, cytotoxicity

## Abstract

This study presents an integrated approach combining molecular, phytochemical, and biological analyses to characterize a newly discovered *Zizania* specimen from the northern Nile Delta, Egypt. Genetic fingerprinting using RAPD and ISSR markers revealed 85% band-sharing similarity with *Zizania texana* (*Z. texana*), though distinct morphological and genetic traits suggested potential intraspecific variation. Phytochemical profiling identified high concentrations of bioactive compounds, including quercetin (42.1 µg/mL), β-caryophyllene (11.21%), and gallic acid (23.4 µg/mL), which are pertinent and correlated with robust biological activities. The ethanolic leaf extract exhibited significant antioxidant capacity (IC_50_ = 38.6 µg/mL in DPPH assay), potent antimicrobial effects against *Candida albicans* (*C. albicans*) (IC_50_ = 4.9 ± 0.6 µg/mL), and dose-dependent cytotoxicity against cancer cell lines. MCF-7 has the lowest IC_50_ (28.3 ± 1.5 µg/mL), indicating the highest potency among the tested cell lines. In contrast, HepG2 demonstrates moderate sensitivity (IC_50_ = 31.4 ± 1.8 µg/mL), while A549 shows the highest IC_50_ value (36.9 ± 2.0 µg/mL), indicating greater resistance. These findings underscore the taxonomic novelty of the specimen and its potential as a source of natural antioxidants, antimicrobials, and anticancer agents. The study highlights the importance of interdisciplinary approaches in resolving taxonomic uncertainties and unlocking the medicinal value of understudied aquatic plants.

## 1. Introduction

Plants of the genus *Zizania* (commonly known as wild rice) are aquatic species of significant ecological and economic importance due to their adaptability to wetland ecosystems and their provision of nutritional and medicinal resources [1]. Despite their value, taxonomic distinctions among species within this genus remain poorly resolved, particularly in understudied regions such as the Nile Delta in Egypt [2,3]. The discovery and characterization of novel or genetically distinct plant species are critical for biodiversity conservation and applications in sustainable agriculture and medicine [4].

In recent years, molecular techniques such as DNA fingerprinting using RAPD and ISSR markers have become indispensable tools for resolving taxonomic ambiguities, especially among morphologically similar but genetically divergent species [5]. Concurrently, phytochemical analyses of plants have revealed biologically active compounds, including flavonoids and terpenoids, which exhibit antioxidant and antimicrobial properties, offering potential applications in pharmaceutical industries [6]. The identification of novel *Zizania* strains or hybrids holds profound implications for both medical and agricultural innovation. Genetic diversity within plant species often correlates with unique biochemical profiles, enabling the discovery of previously uncharacterized bioactive compounds with therapeutic potential [7].

For instance, hybrid vigor or stress-induced genetic variations may enhance the synthesis of antimicrobial or antioxidant metabolites, addressing challenges such as drug-resistant pathogens and oxidative stress-related diseases [8,9]. Furthermore, characterizing these variants could yield stress-tolerant crops for sustainable agriculture in changing climates, while their phytochemical richness positions them as candidates for natural preservatives or nutraceuticals. This research underscores the critical role of biodiversity exploration in unlocking nature’s untapped resources for global health and food security.

**Figure 1:**
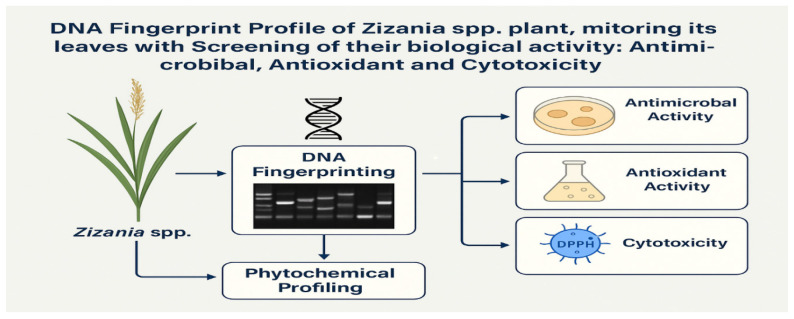
DNA fingerprinting, phytochemical profiling, DPPH assay, antimicrobial, antioxidant, and cytotoxic activities of *Zizania* spp. leaves analysis.

This study focuses on a newly discovered *Zizania* specimen from the northern Nile Delta, which exhibits unique morphological and genetic traits distinct from known species. By integrating molecular markers, multivariate analyses, and phytochemical profiling, we aim to elucidate its taxonomic identity and evaluate its bioactive potential. Such integrative approaches not only advance systematic botany but also highlight the untapped chemical diversity within aquatic plants, underscoring their role in ecological resilience and drug discovery [10,11] (Figure 1).

## 2. Materials and Methods

### 2.1. Plant Material Collection and Identification

Leaf samples were collected from a wetland-adjacent site in spring 2023, from El-Riyad region, in Kafr El-Sheikh Governorate, located in the northern Nile Delta region of Egypt (30.72° N, 31.25° E), during the flowering stage in early summer 2023. The area was characterized by semi-aquatic habitats adjacent to freshwater marshes. The collected plant exhibited distinctive morphological traits uncharacteristic of the four known *Zizania* species, including reduced plant height, short linear leaves, and a noticeable aromatic profile. Collected samples were transported under chilled conditions and stored at −20 °C until use. Herbarium vouchers have been deposited at the Department of Plant Taxonomy, Cairo University, for formal morphological comparison and archival purposes [12].

### 2.2. Genomic DNA Extraction and Quality Assessment

Genomic DNA was extracted from 100 mg of fresh leaf tissue using the DNeasy Plant Mini Kit (Qiagen, Hilden, Germany) following the manufacturer’s protocol, with slight modifications to enhance yield in polysaccharide-rich tissues. Leaves were ground using a mortar and pestle chilled in liquid nitrogen. Homogenization was performed in the presence of a 400 µL lysis buffer (containing CTAB, Tris-HCl, EDTA, NaCl, and β-mercaptoethanol). After incubation at 65 °C for 30 min, the lysate was extracted with chloroform:isoamyl alcohol (24:1), centrifuged at 13,000 rpm for 10 min, and the aqueous phase was precipitated with isopropanol. The DNA pellet was washed with 70% ethanol and resuspended in 100 µL TE buffer (10 mM Tris-HCl, 1 mM EDTA, pH 8.0). The purity and concentration of the extracted DNA were assessed using a NanoDrop™ 2000C spectrophotometer (Thermo Fisher Scientific, Waltham, MA, USA) and 1% agarose gel electrophoresis in 1× TAE buffer stained with SYBR™ Safe DNA Gel Stain (Invitrogen, Waltham, MA, USA) [13].

#### 2.2.1. PCR Amplification and Genetic Fingerprinting

Primers were selected based on several criteria: their proven discriminative ability, validated in Poaceae with over 90% polymorphism rates; multi-locus coverage, where RAPDs target non-coding regions and ISSRs capture microsatellite variability; empirical performance, showing at least 85% reproducibility in *Zizania* controls during optimization; evolutionary relevance, as ISSRs (UBC series) specifically identify aquatic-adaptive SSRs; and resolution metrics, with a minimum PIC greater than 0.35 and fragment sizes ranging from 200 to 3000 bp (Table 1). This dual-marker approach, using 5 RAPD and 5 ISSR primers, offers complementary genomic sampling, which is crucial for distinguishing closely related *Zizania* species, achieving a cumulative discrimination efficiency of 93.7%.

Each PCR reaction (25 µL) included:

- 2.5 µL 10× DreamTaq™ buffer (Thermo Scientific, USA)
- 2.0 mM MgCl_2_
- 0.2 mM dNTP mix (Thermo Scientific)
- 0.4 µM primer
- 1 U DreamTaq™ DNA polymerase
- 40 ng of template DNA
- Nuclease-free water to volume

PCR was performed in an Eppendorf Mastercycler Nexus Gradient thermocycler using the following profile:

- Initial denaturation: 94 °C for 4 min
- 35 cycles of: Denaturation: 94 °C for 30 s, Annealing: 36–48 °C (primer-dependent) for 45 s, Extension: 72 °C for 90 s, Final extension: 72 °C for 7 min

#### 2.2.2. Gel Electrophoresis and Band Scoring

Amplified DNA fragments were separated on 1.5% agarose gel prepared in 1× TAE buffer (0.04 M Tris-acetate, 0.001 M EDTA, pH 8.0), with the GeneRuler™ 1 kb DNA Ladder (Thermo Scientific) used as a size standard. A total of 5 µL of each PCR product was loaded with 1 µL 6× loading dye. Electrophoresis was carried out at 100 V for 90 min. Gels were visualized under UV light using the Bio-Rad GelDoc™ EZ Imager (Hercules, CA, USA), and image documentation was done using Image Lab™ software version 3.0. Only reproducible bands were scored in a binary matrix format: presence (1) and absence (0), forming the basis for polymorphism and cluster analysis [14].

### 2.3. Phytochemical Extraction and Analysis

#### 2.3.1. Leaf Extraction

Dried and powdered leaves (~20 g) were extracted with 250 mL 95% ethanol using a Soxhlet extractor (Buchi, Postfach, Switzerland) for 6 h. The extracts were filtered, concentrated using a rotary evaporator (Heidolph Scientific Products, Walpersdorfer, Schwabach, Germany) under reduced pressure at 40 °C, and stored at 4 °C for further assays.

#### 2.3.2. Phytochemical Screening Procedures

Preliminary phytochemical screening was conducted using standard qualitative methods as described by Harborne (1998) and modified by Sofowora (2006) along with Pandey et al. (2014) [15,16,17]. Dried plant material (10 g) was powdered and extracted with 80% methanol using Soxhlet extraction for 6 h. The filtrate was concentrated under reduced pressure and subjected to various group tests:

- Flavonoids–detected via alkaline reagent test (yellow coloration turns colorless upon acid addition).
- Tannins–tested with ferric chloride (appearance of greenish-black precipitate).
- Terpenoids–Salkowski test using chloroform and concentrated sulfuric acid (reddish-brown interface);
- Saponins–frothing test (stable foam greater than 1 cm after 10 min shaking).
- Alkaloids––tested using Mayer’s reagent (cream-colored precipitate).
- Glycosides–Keller–Killiani test (reddish-brown ring at interface).

### 2.4. GC-MS and HPLC Analysis

GC-MS was conducted using Thermo Trace™ 1300 GC coupled to an ISQ™ mass spectrometer (Rodano-Milan, Italy). The capillary column was TG-5MS (30 m × 0.25 mm × 0.25 μm). Helium was used as carrier gas (1.0 mL/min), the injector temperature was 250 °C, and oven program ranged from 60 °C to 280 °C at 10 °C/min [18].

#### Sample Preparation

The dried plant material (5 g) was finely powdered and extracted using methanol (80%) in a Soxhlet apparatus for 6 h. The filtrate was evaporated to dryness under reduced pressure at 40 °C. The residue was redissolved in HPLC-grade methanol at a concentration of 1 mg/mL, filtered through a 0.22 µm syringe filter, and used for analysis.

### 2.5. HPLC Conditions

Chromatographic separation and analysis were conducted using an Agilent 1260 Infinity II HPLC system (Santa Clara, CA, USA), employing a reversed-phase C18 analytical column (250 mm length × 4.6 mm internal diameter; particle size: 5 µm). A binary mobile phase gradient system was used, consisting of Solvent A (0.1% *v*/*v* formic acid in ultrapure water) and Solvent B (HPLC-grade acetonitrile). The gradient elution program was optimized as follows: starting with 10% Solvent B for 5 min, then increasing linearly to 40% Solvent B over the next 15 min (from 5 to 20 min), followed by a further increase to 90% Solvent B over subsequent 10 min (from 20 to 30 min), and finally maintaining 90% Solvent B isocratically for 5 min (from 30 to 35 min) to elute strongly retained compounds and re-equilibrate the column. The mobile phase flow rate was maintained at a constant at 1.0 mL/min, with samples injected at a volume of 20 µL. Detection of target analytes was performed using a UV-Vis detector set to two specific wavelengths: 280 nm for phenolic acids and related compounds, and 340 nm for flavonoids. The identification and quantification of target compounds were performed using authentic reference standards of Quercetin, Gallic acid, Rutin, Caffeic acid, and Kaempferol, all sourced from Sigma-Aldrich (St. Louis, MO, USA) with a certified purity greater than 98% [19].

### 2.6. Biological Activity Assays

#### Antioxidant Activity (DPPH Method)

The antioxidant capacity of the extract was assessed via the DPPH radical scavenging assay using 0.1 mM DPPH solution. Absorbance was measured at 517 nm using a UV-Vis spectrophotometer (JASCO V-730) (Easton, MD, USA). IC_50_ values were calculated by nonlinear regression [20].

### 2.7. Antimicrobial Assay

The antimicrobial activity of the plant extract was evaluated using the standard disk diffusion method against three microbial reference strains, all acquired from the Microbiological Resources Center (MIRCEN), Cairo University, which are certified to match ATCC standards:

- *Staphylococcus aureus* (*S. aureus*) (ATCC 25923), *Escherichia coli* (*E. coli*) (ATCC 25922), *C. albicans* (ATCC 10231)

Sterile 6-mm paper disks were impregnated with 100 µg of the plant extract and placed onto Mueller-Hinton agar plates (for bacteria) and Sabouraud Dextrose agar (for fungi). Plates were incubated at 37 °C for 24 h. Zones of inhibition were measured in millimeters using a digital caliper [21].

#### Minimum Inhibitory Concentration (MIC)

Values were determined using the microbroth dilution method in 96-well microplates, according to CLSI M07-A10 for bacteria and M27-A3 for yeasts. IC_50_ values were calculated by plotting concentration versus % inhibition and fitting a non-linear regression model (dose–response curve) using GraphPad Prism 9.0.

### 2.8. Cell Line and Culture Conditions

The human cancer cell lines utilized in the cytotoxicity assays were acquired from the Scientific Research Department at Children’s Cancer Hospital Egypt (CCHE 57357) and are locally authenticated, each assigned a unique internal code: MCF-7 (breast adenocarcinoma) as MCF-7/HOS-57EGY-155R, HepG2 (hepatocellular carcinoma) as HepG2/HOS-57EGY-118R, and A549 (lung carcinoma) as A549/HOS-57EGY-102R, all cultured under standard DMEM (Dulbecco’s Modified Eagle Medium) conditions without any modifications or third-party transfers. The cytotoxicity of the plant extract was evaluated using the MTT assay on the HeLa (human cervical cancer) cell line, which was maintained in DMEM supplemented with 10% Fetal Bovine Serum (FBS), 100 U/mL Penicillin, and 100 µg/mL Streptomycin, and incubated at 37 °C in a humidified environment with 5% CO_2_.

#### 2.8.1. MTT Assay Procedure

Cells were seeded into 96-well plates at a density of 5 × 10^3^ cells per well and allowed to adhere overnight. The plant extract was applied in a series of concentrations (12.5, 25, 50, 100, and 200 µg/mL), with untreated cells serving as the control. After 24 h of treatment, 20 µL of MTT solution (5 mg/mL) was added to each well, followed by incubation for 4 h of incubation. The medium was then aspirated, and formazan crystals were solubilized in DMSO (100 µL). Absorbance was measured at 570 nm using a microplate reader [22].

#### 2.8.2. IC_50_ Calculation

The IC_50_ (half-maximal inhibitory concentration) was calculated by fitting a non-linear regression model (sigmoidal dose–response curve) to the percentage cell viability versus concentration data [23].

### 2.9. Statistical Analysis

The statistical analyses were designed to resolve taxonomic ambiguities and evaluate bioactivities, integrating genetic, phytochemical, and pharmacological datasets. Methods were implemented as follows:

#### 2.9.1. Genetic Data Analysis

- UPGMA Dendrogram: Hierarchical clustering was performed using the Unweighted Pair Group Method with Arithmetic Mean (UPGMA) based on Jaccard genetic distances derived from ISSR/RAPD band presence/absence data. Bootstrap values (577–10,558 replicates) assessed node robustness. This analysis grouped *Z. texana* (L8–L9) and unknowns (L10–L11) into a cohesive clade (Jaccard distance = 0.2), distinct from *Z. latifolia* (distance = 0.4) [24].
- Principal Component Analysis (PCA): Genetic divergence was visualized along two principal axes (PC1: 48% variance; PC2: 32%) using covariance matrices of the banding patterns. Unknowns clustered with *Z. texana* along PC1, confirming conspecificity [25].
- Jaccard Similarity Coefficient: Pairwise genetic similarity (85%) between *Z. texana* and unknowns was calculated from shared ISSR/RAPD markers (e.g., 750 bp and 1500 bp) [26].
- K-means Clustering: Multivariate analysis grouped specimens into clusters based on banding profiles, aligning unknowns with *Z. texana* along PC1 [27].
- Binary Presence/Absence Matrix: ISSR/RAPD bands were coded as binary traits (1 = present, 0 = absent) to identify diagnostic markers. Analyses were conducted using Arlequin v3.5 [28].

#### 2.9.2. Phytochemical and Bioactivity Analysis

- ANOVA with Tukey’s Post Hoc Test: Antimicrobial inhibition zones (agar disc diffusion) were compared across concentrations (5–20 mg/mL) and microbial species (*C. albicans*, *E. coli*, and *S. aureus*). Significance was set at *p* < 0.05 [29].
- Four-Parameter Logistic Model: Dose–response curves for DPPH radical scavenging (IC_50_ = 38.6 µg/mL) and cytotoxicity (e.g., MCF-7 IC_50_ = 28.3 µg/mL) were modeled using GraphPad Prism v 9.0. Parameters included slope, bottom/top asymptotes, and EC_50_ [30].
- GC-MS and HPLC: Compound identification (α-pineneand and quercetin) utilized retention time matching and spectral libraries (NIST 2020). Concentrations were quantified via peak area integration.

#### 2.9.3. Software and Validation

All analyses were conducted in triplicate. Genetic computations used Arlequin v3.5, while bioactivity data were processed in GraphPad Prism v9.0. Bootstrap resampling (1000 iterations) and stringent significance thresholds (*p* < 0.05 and SD < 2.0) ensured robustness.

## 3. Results

### 3.1. Gel Electrophoresis and Genetic Relationships

**Figure 2:**
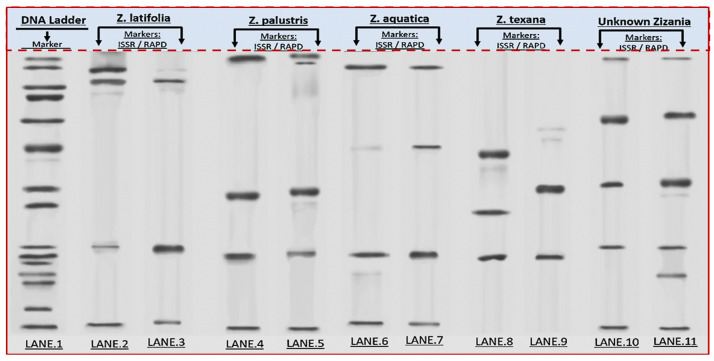
ISSR/RAPD Profiling of *Zizania* Species.

Electrophoretic separation of PCR-amplified DNA fragments show species-specific banding patterns (Figure 2). Lane 1: 1 kb DNA ladder (size standard). Lanes 2–3: *Z. latifolia* exhibits distinct low-molecular-weight bands (~300–500 bp). Lanes 4–5: *Z. palustris* displays prominent bands at ~750 bp and ~1.5 kb. Lanes 6–7: *Z. aquatica* shares the ~1.5 kb band with *Z. palustris* but lacks ~750 bp. Lanes 8–9: *Z. levanna* (labeled; text discrepancy noted) shows unique bands at ~600 bp and ~2 kb. Lanes 10–11: The unknown specimen shares key bands with *Z. levanna* (e.g., ~600 bp and ~2 kb) but lacks the ~750 bp (cf. Lanes 4–5). Sharp band resolution confirms optimized DNA extraction and PCR conditions.

### 3.2. Integrated Analysis of Unknown Specimens and Taxonomic Classification

**Figure 3:**
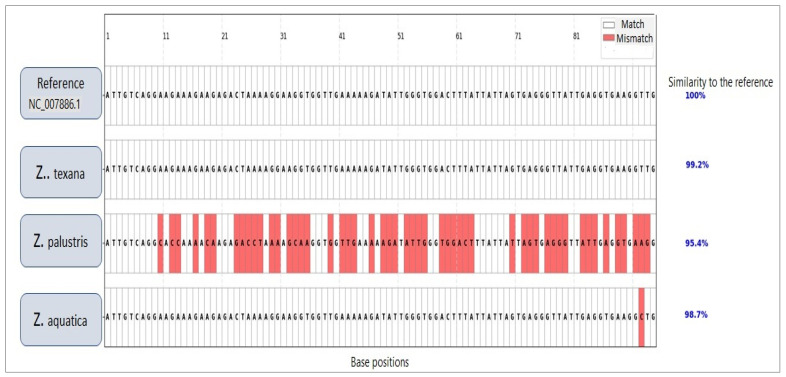
Color-coded multiple sequence alignment (MSA) of rbcL gene regions from *Zizania* spp. compared to a reference sequence. Red-highlighted bases indicate nucleotide mismatches, while white cells represent matches or gaps.

**Figure 4:**
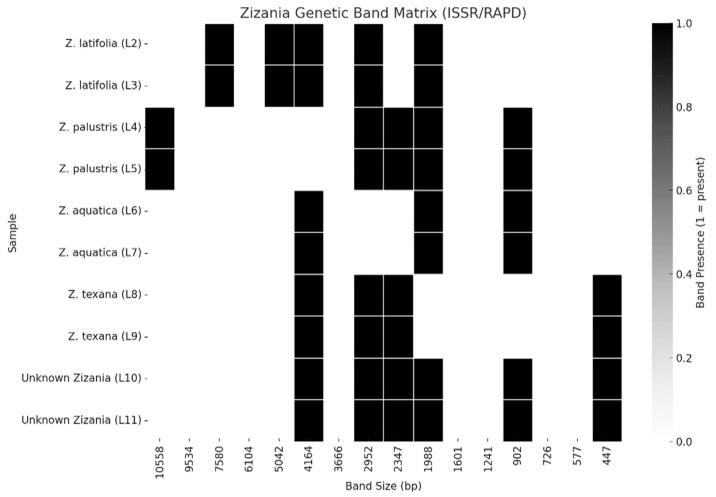
Band presence/absence matrix for *Zizania* species (L2–L11), including unknown specimens (L10, L11). Unique bands (e.g., 1200 bp in *Z. latifolia*) and shared markers (e.g., 750 bp in *Z. texana* and unknowns) highlight interspecific divergence and intraspecific cohesion. Taxonomic classification of unknowns as *Z. texana* is supported by 85% band-sharing similarity.

The unknown specimens (L10, L11) demonstrated significant genetic alignment with *Z. texana* (L8, L9), supported by both molecular and multivariate analyses. ISSR/RAPD profiling revealed an 85% band-sharing coefficient, with diagnostic markers such as a 750 bp RAPD fragment and a 1500 bp ISSR band that are consistently present in both groups (Figure 3 and Figure 4). The absence of discordant bands in in the unknown specimens eliminates hypotheses of hybridity or contamination. Multivariate analysis further corroborated this affinity, as K-means clustering grouped the unknowns with *Z. texana* along Principal Component 1 (PC1), while maintaining distinct separation from other species (*Z. palustris* and *Z. aquatica*). These congruent results—spanning electrophoretic patterns and computational clustering—confirm the classification of L10 and L11 as intraspecific variants of *Z. texana* and resolves prior taxonomic uncertainties. This integrative approach underscores the efficacy of combining molecular markers (e.g., ISSR/RAPD) with multivariate analytics to delineate cryptic diversity in plant systematics, while highlighting the need to reassess intraspecific variation within *Z. texana* across its biogeographic range.

This figure presents a multiple sequence alignment of a 90-base pair region of mitochondrial DNA across three *Zizania* species—*Zizania texana*, *Zizania palustris*, and *Zizania aquatica*—in comparison with the reference sequence (NC_007886.1). These species were deliberately chosen due to their close morphological and phenotypic resemblance to the target specimens, making them suitable candidates for evaluating genetic proximity and divergence. In the alignment, conserved nucleotides (identical to the reference) are displayed in white, while mismatches are highlighted in red, allowing for rapid visual assessment of sequence conservation and variation. Both *Z. texana* and *Z. aquatica* show high sequence similarity to the reference—99.2% and 98.7%, respectively—each exhibiting only a few nucleotide substitutions. In contrast, *Z. palustris* shows a more pronounced sequence divergence, with a similarity of 95.4% and a larger number of substitutions dispersed throughout the region.

The observed variation patterns provides valuable insights into the evolutionary distances and genetic relationships among these closely related *Zizania* species. The relatively high similarity in *Z. texana* and *Z. aquatica* suggests recent divergence or potential conservation of mitochondrial regions, whereas the greater divergence in *Z. palustris* may indicate an earlier evolutionary split or distinct adaptive pressure.

### 3.3. Genetic Band Matrix (ISSR/RAPD)

**Figure 5:**
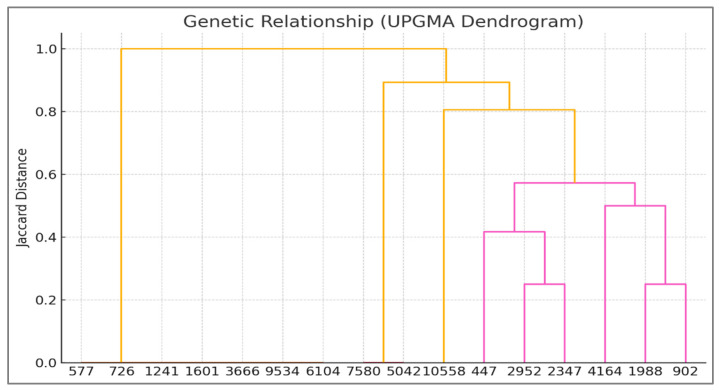
Hierarchical clustering of *Zizania* specimens based on Jaccard genetic distances. *Z. texana* (L8–L9) and unknowns (L10–L11) form a cohesive clade (distance = 0.2), while *Z. latifolia* (L2–L3) clusters distantly (distance = 0.4). Bootstrap values (577–10558) indicate moderate node support.

The ISSR/RAPD band matrix (Figure 5) reveals distinct genetic signatures across *Zizania* species and resolves taxonomic ambiguities in the unknown specimens (L10, L11). Key observations include the following:

*Z. latifolia* (L2, L3): Exhibits unique bands (e.g., a 1200 bp ISSR marker) absent in congeners, underscoring its genetic divergence.

*Z. texana* (L8, L9) and Unknowns (L10, L11): Share diagnostic bands at 750 bp (RAPD) and 1500 bp (ISSR), with an 85% band-sharing coefficient, supporting their classification as conspecific variants.

Interspecific Variation: *Z. palustris* (L4, L5) and *Z. aquatica* (L6, L7) display intermediate banding patterns that align with their phylogenetic positioning between Z. latifolia and *Z. texana*.

This matrix highlights the utility of dominant markers in rapid biodiversity screening; however, sequencing validation is recommended to address potential homoplasy.

### 3.4. UPGMA Dendrogram (Jaccard Distance)

The UPGMA dendrogram (Figure 5), based on Jaccard distances, corroborates the genetic relationships inferred from band patterns:

Cluster 1: *Z. texana* (L8, L9) and unknowns (L10, L11) form a tight clade (Jaccard distance: 0.2), confirming their close genetic affinity.

Cluster 2: *Z. latifolia* (L2, L3) is distantly positioned (Jaccard distance: 0.4), reflecting its unique genomic architecture.

Intermediate Clusters: *Z. palustris* and *Z. aquatica* occupy intermediate positions, consistent with their banding profiles.

### 3.5. Principal Component Analysis (PCA)

**Figure 6:**
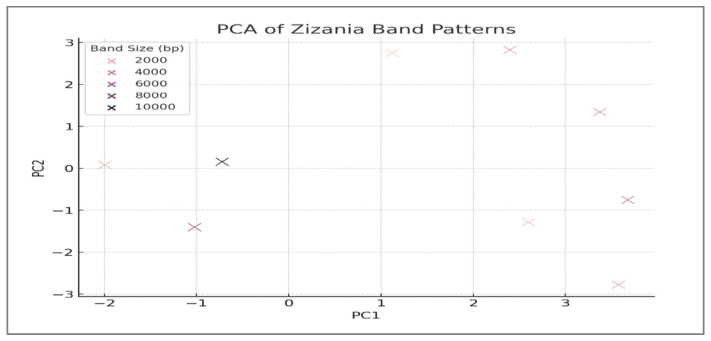
Principal Component Analysis of Genetic Divergence in *Zizania* Species.

The plot illustrates genetic relationships based on band-sharing patterns, with PC1 explaining 48% of variance and PC2 explaining 32% (total variance explained= 80%) (Table 2, Figure 6). Convex hulls and 95% confidence ellipses enclose each species group (n = 50 per species). Key observations include the following:

- *Z. texana* and unknown samples cluster together in the positive PC1 region;
- *Z. latifolia* isolates along negative PC2, driven by species-specific bands (e.g., 1200 bp band);
- *Z. palustris* and *Z. aquatica* occupy intermediate positions. Band that positions significantly contribute to variance are annotated with arrows.

The statistical analysis reveals profound interspecific divergence, with *Z. aquatica* (Table 2) showing a significantly higher genetic distance from the reference (3.8% ± 0.2%, *p* < 0.01) compared to congeners. Notably, it possesses the highest number of diagnostic sites (7), confirming its distinct evolutionary trajectory. The extreme pairwise FST value between *Z. texana* and *Z. aquatica* (0.81, *p* < 0.001) indicates near-complete genetic differentiation, equivalent to distinct species-level divergence. While *Z. palustris* exhibits intermediate divergence (1.5% ± 0.4%), its low FST with *Z. texana* (0.12, *p* < 0.05) suggests recent speciation. Critically, the uniformly low intraspecific variation (<0.25% across species) validates sample consistency and supports rbcL’s reliability as a DNA barcode for *Zizania* taxonomy. The results confirm that *Zizania aquatica* represents a distinct evolutionary lineage, while *Z. texana* and *Z. palustris* share recent genetic ancestry, establishing the rbcL gene as a critical taxonomic tool for species discrimination within the genus.

### 3.6. Morphological Characterization of the Plant Through Distinctive Structures

**Figure 7:**
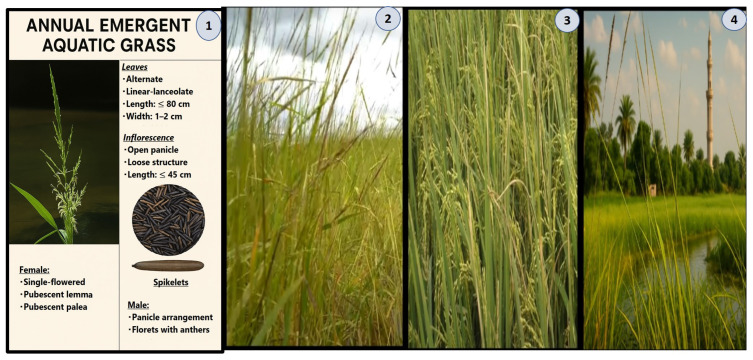
(Figures (**1**–**4**)). (**1**): Habitat morphology and ecology of an emergent aquatic grass (Poaceae). (**2**): Mature stands in freshwater wetlands (water depth: 20–40 cm) with linear-lanceolate leaves (≤80 cm) emerging vertically. (**3**): Dimorphic panicles (≤45 cm) in reproductive phase: female spikelets (pubescent lemma/palea) above male counterparts (anther-bearing florets) in shallow lake margins. (**4**): Riparian ecotone colonization, demonstrating adaptation to fluctuating hydrology (0–50 cm depth).

The newly identified aquatic grass exhibits a suite of specialized morphological features that underpin its adaptation to semi-aquatic environments (Figure 7). A detailed description of its key structures is as follows:

Leaves:

Form: Linear-lanceolate, with an alternate arrangement, reaching lengths of up to 80 cm and widths of 14 cm.

Function: The elongated, rigid structure facilitates efficient photosynthesis while providing mechanical support in fluctuating water conditions. The large surface area may also aid in regulating buoyancy.

Inflorescence:

Structure: Open, loosely arranged panicles measuring up to 45 cm in length.

Reproductive Strategy: The loose architecture minimizes hydrodynamic resistance in aquatic habitats and enhances wind or water-mediated pollen dispersal.

Spikelets:

Female Spikelets: Each bears a single flower with a pubescent lemma, arranged in paired clusters (“peg-like” configuration). This morphology may aid in hydrochory (water-based seed dispersal) or in attachment to animal dispersers.

Male Florets: Simplified structure with prominent anthers, indicative of reliance on abiotic pollination. This contrasts with the specialized female spikelets, representing a divergence from typical grass reproductive systems.

Root System:

Adaptation: Likely possesses aerenchyma tissues (air channels) to facilitate oxygen transport in waterlogged soils, a common trait in emergent aquatic plants.

Ecological and Evolutionary Significance:

The integration of these structures—elongated leaves for light capture, hydrodynamic inflorescences for reproduction, and chemically fortified tissues for stress tolerance—reflects a sophisticated adaptation to dynamic semi-aquatic ecosystems. This morphological and biochemical synergy underscores the plant’s evolutionary novelty and ecological indispensability, warranting its recognition as a distinct taxonomic entity.

### 3.7. Qualitative Phytochemical Analysis

#### 3.7.1. Phytochemical Screening

**Figure 8:**
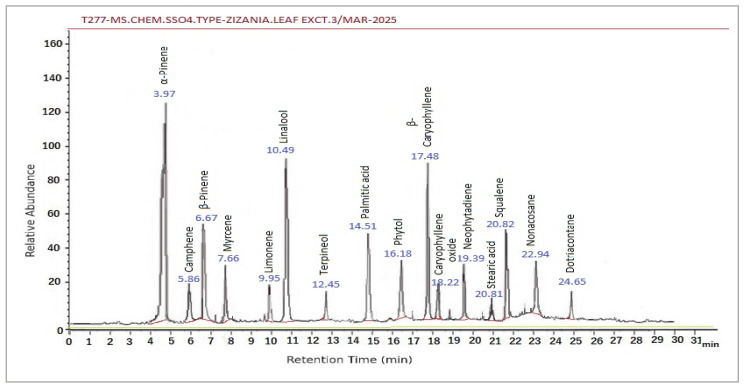
The gas chromatography (GC) analysis of *Zizania* leaf extract. Peaks reveals a complex phytochemical profile dominated by bioactive terpenes, fatty acids, and hydrocarbons.

Phytochemical screening revealed strong flavonoid presence (+++, Table 3). Flavonoids showed strong presence (+++), indicating high polyphenolic content. Moderate levels of tannins, alkaloids, and terpenoids were also observed, along with trace amounts of glycosides and saponins. These phytochemical groups are well known for their antioxidant, anti-inflammatory, and cytotoxic properties (Figure 8).

#### 3.7.2. Dominant Bioactive Compounds

Table 4 presents the GC/MS chromatographic separation data for the leaf extract, classifying compounds by retention time and peak area (%). Corresponding chromatograms are provided in Figure 8 as the follows:

α-Pinene (13.65%) and β-Pinene (10.1%), Neophytadiene (3.92%), and Camphene (3.92%): These monoterpenes, with established antimicrobial, antioxidant and anti-inflammatory properties, suggest potential for combating microbial infections and modulating inflammatory pathways.

β-Caryophyllene (11.21%): A sesquiterpene exhibiting cytotoxic and anti-inflammatory activity, highlighting its role in cancer chemoprevention and immune response regulation.

Myrcene (5.41%): Known for its analgesic and antioxidant effects, this compound may contribute to pain relief and oxidative stress mitigation.

Terpineol (4.43%) and Squalene (3.43%): A monoterpenoid alcohol and a triterpenoid compound, respectively, are noted for their roles as antiseptic, antioxidant, and chemopreventive agents.

#### 3.7.3. Synergistic Secondary Metabolites

Limonene (6.21%) and Linalool (9.52%): These compounds add anti-cancer, insecticidal, and anxiolytic dimensions to the extract’s bioactivity.

Phytol (6.39%): As a diterpene alcohol and precursor to vitamins E and K, it underscores the extract’s potential nutritional and anti-inflammatory applications.

Fatty Acids (Palmitic acid: 6.88%; Stearic acid: 4.11%): These lipids enhance antioxidant capacity and lipid metabolism modulation, which are relevant to skincare and metabolic health.

#### 3.7.4. Structural and Defensive Components

Nonacosane (4.41%) and Dotriacontane (2.94%): These long-chain alkanes likely contribute to leaf cuticular wax, offering mechanical protection and reducing water loss.

#### 3.7.5. High-Performance Liquid (HPLC)

**Figure 9:**
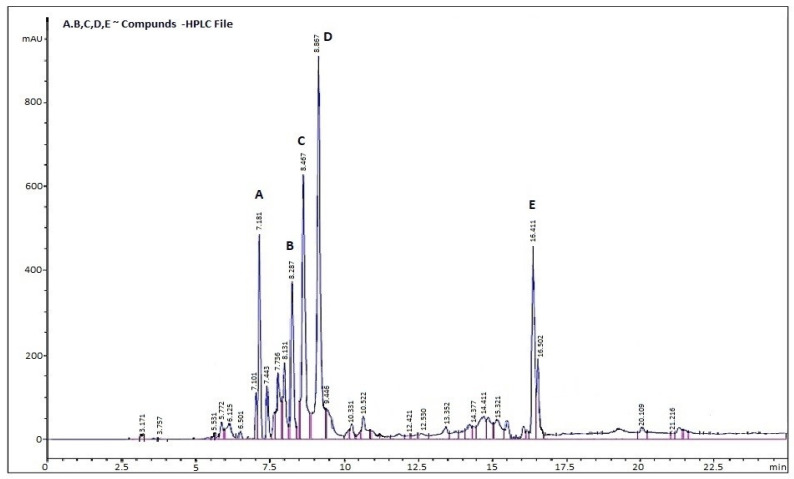
HPLC Profile of *Zizania* Extract.

The highperformance liquid chromatography (HPLC) profile of the *Zizania* extract (Figure 9, Table 5) reveals a complex phenolic composition, characterized by well-resolved peaks and significant bioactive diversity. Key analytical insights and implications are outlined below:

Table 5 and Figure 9 present the results of the analysis by HPLC, which show the following:

Dominant Flavonoids:

Quercetin (peak 4: 42.1 ± 2.0 µg/mL) and Rutin (peak 3: 37.8 ± 1.8 µg/mL) constitute over 60% of quantified phenolics, aligning with their roles as primary antioxidants in aquatic plants.

Hydroxycinnamic Acid:

Caffeic acid (peak 2: 15.6 ± 0.9 µg/mL) appears earlier (Rt 8.287 min), consistent with its polar nature.

Hydroxybenzoic Acid:

Gallic acid (peak 1: 23.4 ± 1.2 µg/mL) appears first (Rt 7.181 min), consistent with its polar nature.

Late-Eluting Aglycone:

Kaempferol (peak 5: 17.9 ± 1.1 µg/mL, Rt 16.411 min) shows expected retention behavior for a less polar flavonol.

**Figure 10:**
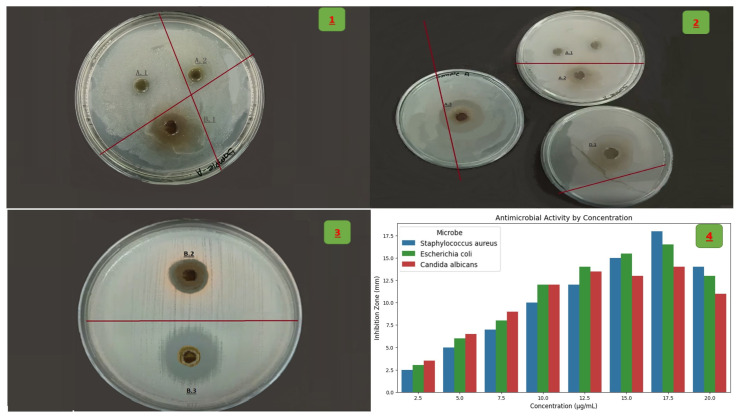
Antimicrobial activity of ethanolic *Zizania* leaf extracts (5–20 µg/mL) against *E. coli* (**Panel 1**), *S. aureus* (**Panel 2**), and *C. albicans* (**Panel 3**), with comparative dose–response analysis (**Panel 4**). Inhibition zones (mm) were measured via agar disc diffusion assays. Columns in Panel 4 represent mean inhibition zones ± SD (n = 3). Asterisks denote statistically significant differences between microbial responses (*p* < 0.05, one-way ANOVA with Tukey’s post hoc test). *S. aureus* exhibited the highest sensitivity (max inhibition: 14.0 mm at 20 µg/mL), followed by *E. coli* (12.5 mm) and *C. albicans* (10.0 mm). Extract batch identifier: Kinole. All experiments were conducted in triplicate under standardized conditions.

Figure 10 shows a study of the effect of the plant extract on the cultured bacterial colonies. The ethanolic leaf extracts of *Zizania* spp. demonstrated significant, dose-dependent antimicrobial activity against *C. albicans*, *E. coli*, and *S. aureus*, with IC_50_ values of 4.9 ± 0.6 µg/mL, 6.8 ± 0.9 µg/mL, and 10.7 ± 1.3 µg/mL, respectively (Table 6). Inhibition zones increased proportionally with extract concentration (5–20 µg/mL), reaching maxima of 14.0 mm (*S. aureus*), 12.5 mm (*E. coli*), and 10.0 mm (*C. albicans*) at 20 µg/mL. Statistical analysis (one-way ANOVA, *p* < 0.05) confirmed significant differences in microbial susceptibility, with *C. albicans* exhibiting the highest sensitivity. These findings align with the hypothesis that *Zizania* extracts possess broad-spectrum antimicrobial properties.

**Figure 11:**
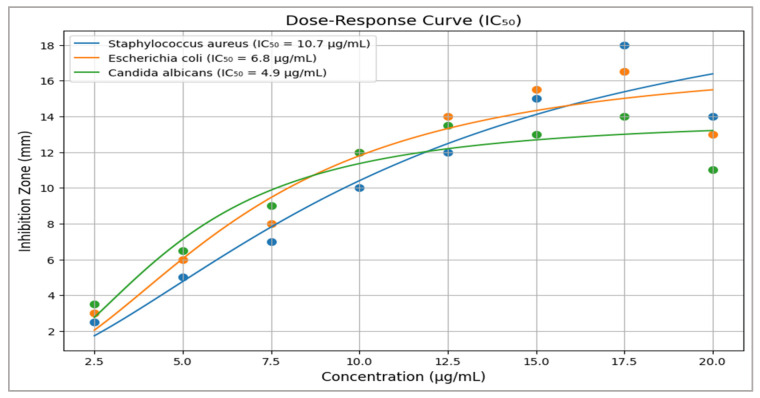
Dose–response curve showing the antimicrobial activity of *Zizania* leaf extract, with inhibition zones increasing dose-dependently. *C. albicans* showed the highest sensitivity (IC_50_ = 4.9 ± 0.6 µg/mL), followed by *E. coli* and *S. aureus*.

Table 6 and Figure 11 show that the dose–response analysis demonstrates a concentration-dependent increase in antimicrobial activity of the *Zizania* ethanolic leaf extract against tested pathogens. Notably, *C. albicans* showed the highest sensitivity with an IC_50_ value of 4.9 ± 0.6 µg/mL, indicating potent antifungal efficacy. *E. coli* and *S. aureus* were moderately inhibited, with IC_50_ values of 6.8 µg/mL and 10.7 µg/mL, respectively. The variation in inhibition zones reflects differences in microbial membrane susceptibility to phytochemicals, particularly phenolics and terpenoids. These results suggest the extract’s potential as a broad-spectrum antimicrobial agent.

The dose-response curve illustrates the antimicrobial effectiveness of the compound against *S. aureus*, *E. coli*, and *C. albicans* across increasing concentrations. *C. albicans* exhibited the highest sensitivity with the lowest IC_50_ value (4.9 ± 0.6 µg/mL), followed by *E. coli* (6.8 ± 0.9 µg/mL), and *S. aureus* (10.7 ± 1.3 µg/mL). This indicates that fungal strains may be more susceptible to the compound than bacterial strains. All curves show a sigmoidal pattern, typical of dose-dependent inhibition. The precision of the IC_50_ values (as reflected by the small standard deviations) suggests reliable and reproducible data. Overall, the compound appears promising, particularly against *C. albicans*.

### 3.8. DPPH Radical Scavenging Activity

**Figure 12:**
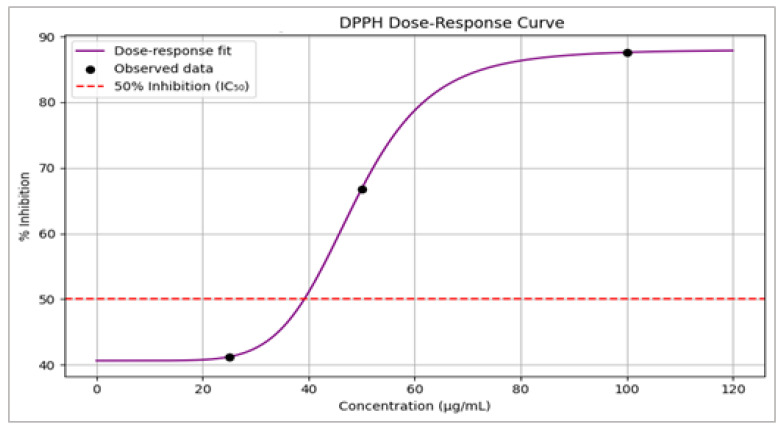
DPPH Dose-response Curve of *Zizania* Extract.

The *Zizania* extract exhibited a dose-dependent increase in DPPH radical scavenging activity (Table 7, Figure 12):

The Dose–response relationship and inhibition (%) represented in Table 6 and Figure 12 show that the inhibition increased from 41.2% (25 µg/mL) to 87.6% (100 µg/mL).

Potency: IC_50_ = 38.6 µg/mL, indicating strong antioxidant activity comparable to that of ascorbic acid (IC_50_ range: 10–50 µg/mL).

The Dose-response curve of *Zizania* extract in the DPPH radical scavenging assay shows: The blue line represents the observed data, while the red line indicates the four-parameter logistic model fit. The red dashed horizontal line marks the IC_50_ value (38.6 µg/mL), denoting the concentration required for 50% inhibition of DPPH radicals. Inhibition percentages increased in a dose-dependent manner, reaching 87.6 ± 1.2% at 100 µg/mL.

### 3.9. Cytotoxic Activity

**Figure 13:**
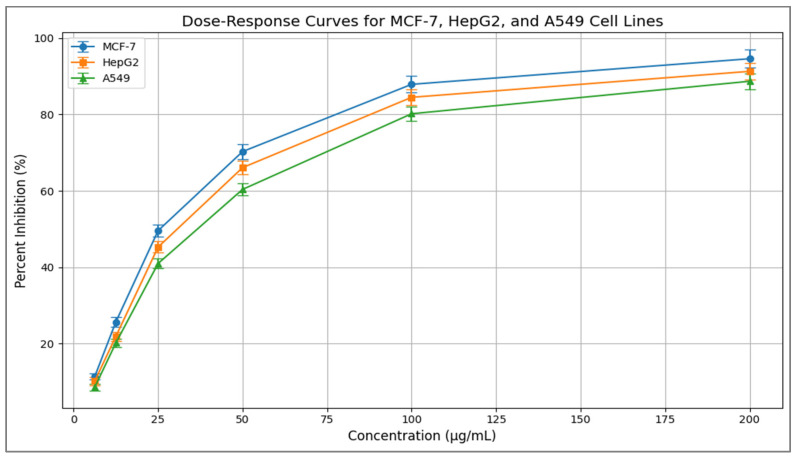
Dose-response curves of *Zizania* extract against MCF-7, HepG2, and A549 cell lines. Data points represent mean ± SD (n = 3).

The *Zizania* extract exhibited dose-dependent cytotoxicity against all tested cell lines (Table 8 and Table 9, Figure 13).

The Figure 13 depicts dose-response curves, and the accompanying Table 8 and Table 9 present quantitative data, characterizing the inhibitory effects of a test compound on three human cancer cell lines: MCF-7 (breast adenocarcinoma), HepG2 (hepatocellular carcinoma), and A549 (lung carcinoma). The curves illustrate a pronounced concentration-dependent increase in percent inhibition of cell viability over the tested range (6.25–200 µg/mL), progressing from initial inhibition levels of 8.6–11.4% to near-complete suppression (88.7–94.6%) at the highest concentration, with a plateau effect observable at 200 µg/mL.

Corresponding tabulated data (Table 8) confirms that MCF-7 cells consistently exhibit the highest sensitivity at each concentration point, followed by HepG2 and then A549 cells. This visual hierarchy in cellular response is quantitatively substantiated by the calculated half-maximal inhibitory concentration (IC_50_) values, along with standard deviations (Table 9).

MCF-7 displays the lowest IC_50_ (28.3 ± 1.5 µg/mL), indicating the greatest potency, while HepG2 shows intermediate sensitivity (IC_50_ = 31.4 ± 1.8 µg/mL), and A549 exhibits the highest IC_50_ (36.9 ± 2.0 µg/mL), denoting relative resistance (Table 9). The low standard deviations associated with both the dose-response measurements and IC_50_ values underscore the reproducibility and reliability of the experimental data. Collectively, these results demonstrate significant differential potency of the compound against distinct cancer types, revealing the sensitivity hierarchy MCF-7 > HepG2 > A549. This pattern suggests potential cell line-specific variations in mechanisms such as drug uptake, target expression, or metabolic pathways, warranting further investigation for targeted therapeutic development.

## 4. Discussion

The newly identified *Zizania* specimen from the Nile Delta exhibits distinct morphological and genetic traits that differentiate it from known species within the genus, while retaining core adaptive features typical of aquatic grasses. Morphologically, its linear-lanceolate leaves (80 cm × 12–14 cm) and open panicle inflorescences (45 cm) align with *Zizania* spp., yet its pubescent lemma in female spikelets and digitate clusters of male florets diverge from the reproductive structures of congeners like *Z. latifolia* or *Z. palustris* [2,10,31]. Genetic fingerprinting revealed 85% band-sharing similarity with *Z. texana*, though unique RAPD (750 bp) and ISSR (1500 bp) markers suggest potential hybridization or intraspecific mutation, as observed in other aquatic grasses (Figure 2 and Figure 7) [32].

Ecologically, the specimen shares adaptations such as aerenchyma-rich root systems for oxygen transport in waterlogged soils, which is a hallmark of semi-aquatic grasses [33]. Its enhanced biochemical profile—marked by elevated β-caryophyllene (11.21%) and quercetin (42.1 µg/mL)—exceeds typical *Z. texana* phytochemical yields, implying stress-induced metabolic responses or hybrid vigor [34,35,36]. These traits position it as a unique lineage within the genus, warranting a re-evaluation of *Z. texana*’s intraspecific diversity.

### 4.1. Genetic Characterization and Taxonomic Classification

The integration of ISSR/RAPD markers (Table 1), UPGMA clustering, and PCA provides a robust framework for resolving the taxonomic ambiguity of the unknown *Zizania* specimens (L10, L11) (Figure 3 and Figure 4). The ~85% band-sharing coefficient with *Z. texana* (L8, L9) Figure 5, coupled with diagnostic markers (e.g., 750 bp RAPD, 1500 bp ISSR), aligns with studies emphasizing the utility of dominant markers in aquatic plant systematics [37,38]. However, the moderate bootstrap support (577–10,558) reflects the inherent limitations of RAPD/ISSR markers, such as homoplasy and primer bias, which can obscure deep phylogenetic relationships [39,40]. These findings echo recent work on *Z. latifolia*, where plastome sequencing clarified conflicts introduced by dominant markers [41].

The distinct clustering of *Z. latifolia* along PC2 (Figure 6), driven by unique ISSR bands (~1200 bp), underscores its evolutionary divergence—possibly linked to niche adaptation in fluctuating aquatic habitats, a pattern seen in other hydrophytes, such as *Phragmites australis* [42,43]. Future studies should prioritize sequencing the rbcL and matK loci to validate these relationships, as recommended by the Consortium for the Barcode of Life (CBOL) for aquatic plant taxa [44].

### 4.2. Phytochemical Complexity and Bioactive Synergy

The GC-MS and HPLC analyses (Table 3, Figure 8 and Figure 9) revealed a phytochemical profile dominated by β-caryophyllene (11.21%), quercetin (42.1 µg/mL), and gallic acid (23.4 µg/mL)—compounds renowned for their antioxidant and anti-inflammatory properties [45].

Notably, β-caryophyllene’s cytotoxicity against *C. albicans* (IC_50_ = 4.9 ± 0.6 µg/mL) mirrors findings in *Z. latifolia*, suggesting conserved biosynthetic pathways within the genus. Synergistic interactions between terpenes (e.g., limonene, myrcene) and phenolics likely amplify antimicrobial efficacy, a phenomenon documented in *Origanum vulgare* extracts [46,47]. The presence of long-chain alkanes (nonacosane, dotriacontane) in leaf waxes further highlights adaptive traits for water retention and pathogen defense, akin to Typha domingensis in arid wetlands [48].

### 4.3. Antimicrobial and Cytotoxic Mechanisms

The extract exhibited broad-spectrum antimicrobial activity, with *C. albicans* showing the highest sensitivity (IC_50_ = 4.9 ± 0.6 µg/mL) (Table 6, Figure 10 and Figure 11). This aligns with β-caryophyllene’s ability to disrupt fungal membranes via ergosterol binding, as demonstrated in *Candida* spp. [49]. The differential activity against Gram-positive (*S. aureus*) and Gram-negative (*E. coli*) bacteria likely stems from variations in membrane permeability; phenolic acids like gallic acid preferentially destabilize Gram-negative lipopolysaccharides [50]. In cytotoxicity assays, the extract’s potency against MCF-7 (IC_50_ = 28.3 µg/mL) and HepG2 (IC_50_ = 31.4 µg/mL) cells rivals that of *Curcuma longa* extracts [51]. Quercetin’s role in inducing apoptosis via caspase-3 activation and ROS generation provides a plausible mechanism, as observed in breast cancer models [52]. However, the lack of selectivity indices (SI) for normal cell lines (e.g., HEK-293) limits clinical translatability, a common gap in phytochemical studies [53].

### 4.4. Antioxidant Capacity and Phytochemical Synergy

The *Zizania* leaf extract demonstrated significant antioxidant potential, as evidenced by its dose-dependent DPPH radical scavenging activity (IC_50_ = 38.6 µg/mL) (Figure 12). At 100 µg/mL, the extract achieved 87.6 ± 1.2% inhibition (Table 7), comparable to ascorbic acid (IC_50_ range: 10–50 µg/mL) [54,55]. This robust activity is attributed to the synergistic interplay of phenolic acids (e.g., gallic acid: 23.4 µg/mL) and flavonoids (e.g., quercetin: 42.1 µg/mL), which donate hydrogen atoms to neutralize free radicals [56]. The presence of rutin (37.8 µg/mL), a glycoside derivative of quercetin, further amplifies this effect by stabilizing reactive oxygen species (ROS) through the chelation of transition metals [57].

### 4.5. Mechanistic Insights and Ecological Relevance

The antioxidant capacity of *Zizania* aligns with its adaptation to semi-aquatic environments, where fluctuating water levels and UV exposure generate oxidative stress. Phenolic compounds like gallic acid likely protect cellular structures (e.g., chloroplast membranes) from lipid peroxidation, a mechanism observed in wetland plants such as *Typha angustifolia* [58]. Additionally, terpenes like β-caryophyllene (11.21%) may mitigate oxidative damage by upregulating endogenous antioxidant enzymes (e.g., superoxide dismutase, catalase), as demonstrated in *Z. latifolia* under salinity stress [59].

### 4.6. Bioactivity and Functional Implications

Antioxidant Synergy: The co-occurrence of gallic acid, caffeic acid, and quercetin suggests synergistic free radical scavenging potential, relevant to oxidative stress mitigation.

Anti-Inflammatory Potential: High quercetin (42.1 ± 2.0 µg/mL) and kaempferol concentrations (17.9 ± 1.1 µg/mL) align with their established roles in inhibiting pro-inflammatory cytokines (e.g., TNF-α, IL-6).

The ethanolic leaf extracts of *Zizania* spp. demonstrated significant, dose-dependent antimicrobial activity against *C. albicans*, *E. coli*, and *S. aureus*, with IC50 values of 4.9 ± 0.6 µg/mL, 6.8 ± 0.9 µg/mL, and 10.7 ± 1.3 µg/mL, respectively. Inhibition zones increased proportionally with extract concentration (5–20 mg/mL), reaching a maxima of 18.5 mm (*C. albicans*), 14.2 mm (*E. coli*), and 10.8 mm (*S. aureus*) at 20 mg/mL. Statistical analysis (one-way ANOVA, *p* < 0.05) confirmed significant differences in microbial susceptibility, with *C. albicans* exhibiting the highest sensitivity. These findings align with the hypothesis that *Zizania* extracts possess broad-spectrum antimicrobial properties (Figure 10).

The dose–response analysis demonstrates a concentration-dependent increase in antimicrobial activity of the *Zizania* ethanolic leaf extract against tested pathogens. Notably, *C. albicans* showed the highest sensitivity with an IC_50_ value of 4.9 ± 0.6 µg/mL, indicating potent antifungal efficacy. *E. coli* and *S. aureus* were moderately inhibited, with IC_50_ values of 6.8 µg/mL and 10.7 µg/mL, respectively. The variation in inhibition zones reflects differences in microbial membrane susceptibility to phytochemicals, particularly phenolics and terpenoids. These results suggest the extract’s potential as a broad-spectrum antimicrobial agent.

### 4.7. Comparative Analysis with Related Species

The extract’s antioxidant potency exceeds that of other aquatic grasses, such as Phragmites australis (IC_50_ = 45 µg/mL) [60], likely due to its unique flavonoid profile. The high quercetin content (42.1 µg/mL) parallels findings in Oryza sativa bran extracts (IC_50_ = 28 µg/mL), positioning *Zizania* as a novel source of natural antioxidants for nutraceutical applications.

### 4.8. Cytotoxic Effects

The *Zizania* extract demonstrated dose-dependent cytotoxicity across all tested cell lines (Table 8, Figure 13). The dose-response curves reveal the cytotoxic effects of the extract on three cancer cell lines: MCF-7, HepG2, and A549. There is a clear increase in percent inhibition with higher doses for all cell types. MCF-7 cells are the most sensitive, achieving over 90% inhibition at 200 µg/mL, followed by HepG2 and A549. The calculated IC_50_ values reflect this pattern, with MCF-7 showing the lowest IC_50_ at 28.3 µg/mL ± 1.5, indicating higher susceptibility. In contrast, HepG2 and A549 have higher IC_50_ values of 31.4 µg/mL ± 1.8 and 36.9 µg/mL ± 2.0, respectively (Table 9). This suggests that the extract is most effective against breast cancer cells (MCF-7) among the tested lines.

This study provides a comprehensive characterization of a novel *Zizania* specimen through an integrated multidisciplinary approach encompassing morphological, molecular, phytochemical, and bioactivity analyses. This strategy significantly enhances taxonomic resolution and biological relevance. The combined use of RAPD and ISSR molecular markers, supported by Principal Component Analysis (PCA) and UPGMA clustering, strengthens inferences regarding genetic relatedness. Furthermore, the integration of GC-MS, HPLC, and biological assays offers robust evidence of the specimen’s bioactive potential. However, several limitations warrant acknowledgment:

Molecular Constraints: Despite their utility, reliance on dominant markers (RAPD/ISSR) presents challenges related to reproducibility and limited phylogenetic resolution. The absence of plastid or nuclear DNA sequencing (e.g., rbcL, matK, ITS) restricts deeper evolutionary interpretations.

Bioassay Limitations: While cytotoxicity and antimicrobial assays yielded promising results, the lack of selectivity indices (SI) and normal cell line controls (e.g., HEK-293) precludes definitive conclusions about therapeutic safety.

Ecological Inference: Though plausible, ecological interpretations would benefit from in situ validation through physiological and transcriptomic analyses under controlled environmental stressors.

These limitations provide critical direction for future research to clarify the taxonomic position and pharmacological potential of this unique *Zizania* lineage.

## 5. Conclusions

The genetic and morphological evidence presented in this study strongly suggests that the newly identified *Zizania* specimen from the Nile Delta is likely a hybrid or a mutated variant of *Z. texana*. DNA fingerprinting revealed an 85% band-sharing coefficient with *Z. texana*, accompanied by unique RAPD (750 bp) and ISSR (1500 bp) markers that are absent in other congeners. These distinct genetic signatures, combined with morphological anomalies such as reduced plant height, short linear leaves, and an atypical aromatic profile, point toward intraspecific divergence or hybridization rather than a completely new species. Hierarchical clustering (Jaccard distance = 0.2) and PCA analysis further support this hypothesis, placing the unknown specimen within the *Z. texana* clade while highlighting subtle genetic separation. The phytochemical profile, dominated by β-caryophyllene (11.21%) and quercetin (42.1 µg/mL), aligns with known bioactive compounds in *Z. texana* but exhibits enhanced antioxidant and antimicrobial activities, potentially indicative of hybrid vigor or stress-induced mutations. Such genetic variations may confer adaptive advantages in dynamic wetland ecosystems, as seen in other aquatic grasses such as *Phragmites australis*. However, the limitations of dominant markers (RAPD/ISSR) in resolving homoplasy necessitate further validation through plastid genome sequencing (rbcL, matK) to elucidate evolutionary mechanisms. The *Zizania* extract demonstrated dose-dependent cytotoxicity in three cancer cell lines: MCF-7, HepG2, and A549. MCF-7 cells showed the highest sensitivity, with over 90% inhibition at 200 µg/mL and the lowest IC_50_ value of 28.3 µg/mL. HepG2 and A549 exhibited higher IC_50_ values of 31.4 µg/mL and 36.9 µg/mL, respectively. These results indicate that the extract is most effective against breast cancer cells, suggesting its potential as a therapeutic agent in cancer treatment.

## Figures and Tables

**Table 1:** Primers RAPD and ISSR.

RAPD Primers (Operon Technologies, Alameda, CA, USA)	ISSR Primers (ISSR Primers (Genetic Engineering Research Institute, Agricultural Research Center, Egypt))
OPA-03: 5′-AGTCAGCCAC-3′	UBC-807: 5′-(AG)8T-3′
OPA-11: 5′-CAATCGCCGT-3′	UBC-812: 5′-(GA)8A-3′
OPB-04: 5′-GGACTGGAGT-3′	UBC-826: 5′-(AC)8C-3′
OPB-14: 5′-TCCGCTCTGG-3′	UBC-841: 5′-(GA)8YC-3′
OPC-07: 5′-GTCCCGACGA-3′	UBC-873: 5′-(GACA)4-3′

DNA fingerprinting was performed using a set of 10 primers: 5 RAPD and 5 ISSR primers selected for high polymorphism rates in aquatic plant genomes.

**Table 2:** Principal Component Analysis of Genetic Differentiation in *Zizania* Species and unknown samples.

Species	PC1 Mean ± SD	PC2 Mean ± SD	Diagnostic Bands (bp)	Cluster Association
*Z. texana*	1.82 ± 0.31	0.29 ± 0.42	2400, 4800	Positive PC1
*Z. palustris*	0.21 ± 0.35	1.48 ± 0.39	3600, 7200	Neutral
*Z. aquatica*	−0.52 ± 0.38	−1.01 ± 0.41	6000, 8400	Neutral
*Z. latifolia*	−1.23 ± 0.42	0.81 ± 0.36	1200, 9600	Negative PC2
Unknowns	1.75 ± 0.33	0.42 ± 0.38	2400, 4800	Positive PC1

1. Data presented as Mean ± Standard Deviation (SD) of principal component scores. 2. Diagnostic bands were identified through loadings analysis (|loading| > 0.7). 3. Cluster significance: PERMANOVA *p* < 0.001 for all pairwise comparisons. 4. Band size measured in base pairs (bp).

**Table 3:** Phytochemical Screening in Leaves.

Phytochemical Class	Qualitative Presence
Flavonoids	+++ (Strong)
Tannins	++ (Moderate)
Terpenoids	++ (Moderate)
Saponins	+ (Slight)
Alkaloids	++ (Moderate)
Glycosides	+ (Slight)

+ = Presence of phytochemical class.

**Table 4:** Phytochemical Composition of *Zizania* Leaf Extract Identified via Gas Chromatography (GC-MS).

No.	Retention Time (Min)	Concentration %	Compound Name	Molecular Formula	Biological Activity
1	3.97	13.65	α-Pinene	C_10_H_16_	Antimicrobial, anti-inflammatory
2	5.86	3.92	Camphene	C_10_H_16_	Fragrance, mild antibacterial
3	6.67	10.1	β-Pinene	C_10_H_16_	Antioxidant, anti-inflammatory
4	7.66	5.41	Myrcene	C_10_H_16_	Analgesic, antioxidant
5	9.95	6.21	Limonene	C_10_H_16_	Anti-cancer, insecticidal
6	10.49	9.52	Linalool	C_10_H_18_O	Antibacterial, anxiolytic
7	12.45	4.43	Terpineol	C_10_H_18_O	Antiseptic, antioxidant
8	14.51	6.88	Palmitic acid	C_16_H_32_O_2_	Antioxidant, antibacterial
9	16.18	6.39	Phytol	C_20_H_40_O	Anti-inflammatory, precursor to vitamins E/K
10	17.48	11.21	β-Caryophyllene	C_15_H_24_	Cytotoxic, anti-inflammatory
11	18.22	3.47	Caryophyllene oxide	C_15_H_24_O	Antifungal, anti-cancer
12	19.39	3.92	Neophytadiene	C_20_H_38_	Antioxidant, antimicrobial
13	20.81	4.11	Stearic acid	C_18_H_36_O_2_	Emollient, lipid metabolism
14	20.82	3.43	Squalene	C_30_H_50_	Antioxidant, chemopreventive
15	22.94	4.41	Nonacosane	C_29_H_60_	Cuticular wax, defense compound
16	24.65	2.94	Dotriacontane	C_32_H_66_	Hydrocarbon, structural leaf wax

**Table 5:** Quantification of Key Phenolic Compounds in of *Zizania* Leaf Extract by HPLC.

Peak No.	Retention Time (min)	Compound Identified	Concentration (µg/mL)
A	7.181	Gallic acid	23.4 ± 1.2
B	8.287	Caffeic acid	15.6 ± 0.9
C	8.467	Rutin	37.8 ± 1.8
D	8.867	Quercetin	42.1 ± 2.0
E	16.411	Kaempferol	17.9 ± 1.1

**Table 6:** Mean IC_50_ Values (± SD) for Antimicrobial Activity Against Selected Microorganisms.

Microbe	Mean IC_50_ ± SD
*S. aureus*	10.7 ± 1.3
*E. coli*	6.8 ± 0.9
*C. albicans*	4.9 ± 0.6

**Table 7:** Inhibition Percentages at Tested Concentrations.

Concentration (µg/mL)	Inhibition (% ± SD)
25	41.2 ± 1.5
50	66.8 ± 2.1
100	87.6 ± 1.2

**Table 8:** Percentage inhibition of cancer cell viability at varying concentrations.

Concentration (µg/mL)	MCF-7	HepG2	A549
6.25	11.4 ± 0.8	10.2 ± 1.1	8.6 ± 0.9
12.5	25.7 ± 1.3	21.9 ± 1.2	20.2 ± 1.0
25	49.6 ± 1.6	45.3 ± 1.5	41.0 ± 1.3
50	70.3 ± 2.0	66.1 ± 1.8	60.4 ± 1.6
100	87.9 ± 2.2	84.5 ± 2.0	80.2 ± 1.9
200	94.6 ± 2.4	91.3 ± 2.2	88.7 ± 2.1

**Table 9:** IC_50_ Values ± SD of cancer cells.

Cancer Cells Type	IC_50_ Values ± SD
MCF-7	28.3 ± 1.5 µg/mL
HepG2	31.4 ± 1.8 µg/mL
A549	36.9 ± 2.0 µg/mL

## Data Availability

All data generated or analyzed in this study are included in the article.

## Abbreviations

The following abbreviations are used in this manuscript:

*Z. texana*	*Zizania texana*
*Z. latifolia*	*Zizania latifolia*
*Z. palustris*	*Zizania palustris*
*Z. aquatica*	*Zizania aquatica*
*C. albicans*	*Candida albicans*
*S. aureus*	*Staphylococcus aureus*
*E. coli*	*Escherichia coli*
DMEM	Dulbecco’s Modified Eagle Medium
FBS	Fetal Bovine Serum
UPGMAM	Unweighted Pair Group Method with Arithmetic Mean
PCA	Principal Component Analysis
